# A Zebrafish Drug-Repurposing Screen Reveals sGC-Dependent and sGC-Independent Pro-Inflammatory Activities of Nitric Oxide

**DOI:** 10.1371/journal.pone.0137286

**Published:** 2015-10-07

**Authors:** Christine Wittmann, Markus Reischl, Asmi H. Shah, Eva Kronfuss, Ralf Mikut, Urban Liebel, Clemens Grabher

**Affiliations:** 1 Institute of Toxicology and Genetics, Karlsruhe Institute of Technology, Eggenstein-Leopoldshafen, Germany; 2 Institute for Applied Computer Science, Karlsruhe Institute of Technology, Eggenstein-Leopoldshafen, Germany; National University of Singapore, SINGAPORE

## Abstract

Tissue injury and infection trigger innate immune responses. However, dysregulation may result in chronic inflammation and is commonly treated with corticosteroids and non-steroidal anti-inflammatory drugs. Unfortunately, long-term administration of both therapeutic classes can cause unwanted side effects. To identify alternative immune-modulatory compounds we have previously established a novel screening method using zebrafish larvae. Using this method we here present results of an *in vivo* high-content drug-repurposing screen, identifying 63 potent anti-inflammatory drugs that are in clinical use for other indications. Our approach reveals a novel pro-inflammatory role of nitric oxide. Nitric oxide affects leukocyte recruitment upon peripheral sensory nervous system or epithelial injury in zebrafish larvae both via soluble guanylate cyclase and in a soluble guanylate cyclase -independent manner through protein S-nitrosylation. Together, we show that our screening method can help to identify novel immune-modulatory activities and provide new mechanistic insights into the regulation of inflammatory processes.

## Introduction

Inflammation is a highly regulated response of the innate immune system in reaction to tissue injury or infection. However, its dysregulation may lead to chronic inflammatory states with pathological consequences including autoimmune and neurodegenerative diseases [1]. Current interventional therapies rely on corticosteroids and non-steroidal anti-inflammatory drugs (NSAIDs) based on inhibiting the synthesis or action of inflammatory mediators. While corticosteroids induce general immune suppression, NSAIDs cause symptomatic relief by counteracting vasodilation, reducing fever and suppression of inflammatory pain [2]. However, long-term administration of corticosteroids and NSAIDs often leads to unwanted side effects including immunodeficiency, hyperglycemia or osteoporosis [3]. Thus, there is a demand for alternative therapeutics with higher specificity and less side effects [4].

Drug repurposing, the testing of approved drugs for new off-label effects, promises to accelerate the period from drug discovery to clinical use. In recent years the zebrafish emerged as a particularly versatile vertebrate model organism for drug repositioning screens [5]. Due to their small size and relative ease of manipulation and observation, zebrafish embryos and larvae are perfectly suited for small molecule screens in a whole-animal context [6]. In line with these notions, we recently developed a prototype automated high-content screening approach in zebrafish that allows rapid phenotypic identification of immune-modulatory activities through chemical induction (ChIn) of tissue injury within the peripheral sensory nervous system based on copper sulfate (CuSO_4_) treatment [7]. CuSO_4_ causes oxidative stress in hair cells of lateral line neuromasts followed by hair cell death. This initiates an acute inflammatory response, which is characterized by rapid recruitment of leukocytes to injured neuromasts. Fluorescent reporter expression in both leukocytes and neuromasts allows computer-aided automated signal detection and quantification of the inflammatory response. Here we further refined this assay and report the results of an automated ChIn–based screen of 1120 drugs identifying 63 potent anti-inflammatory hits from diverse pharmacological classes, most of which were previously not associated with immune modulatory activity.

## Results and Discussion

### An automated phenotypic screen for immune modulatory compounds in zebrafish

**Fig 1:**
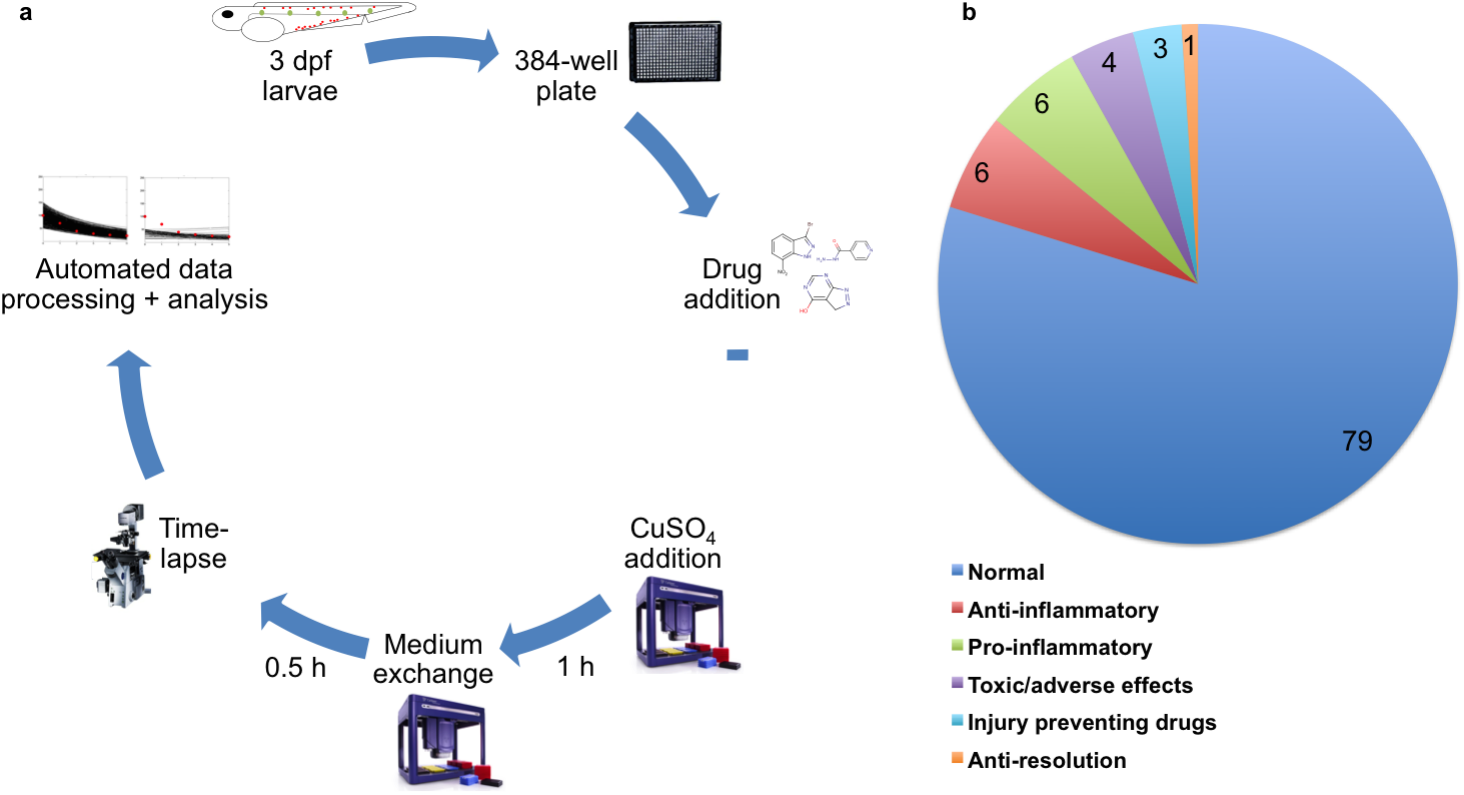
Experimental setup and compound categorization of the automated ChIn screen. **(a)** Schematic diagram illustrating the screening strategy for compounds modulating inflammation. Individual larvae are distributed in 384-well plates. Drugs are added 1 h prior to treatment with CuSO_4_ for 1h at 28°C in the dark followed by an exchange of medium and a 6h time-lapse analysis. Finally, screening data are automatically processed and analyzed. **(b)** Pie chart displaying the identified compound categories upon screening 1120 compounds of an FDA-approved library and the ICCB library of known bioactives. Numbers indicate percentages.

We devised a fully automated screening strategy to screen small molecule libraries for immune modulatory activities in zebrafish larvae (Fig 1A). The automated ChIn assay is a multi-parametric assay capable of identifying not only compounds reducing (anti-inflammatory) or exaggerating inflammation (pro-inflammatory), but also compounds affecting inflammation resolution as well as providing information on compound toxicity and off-target effects.

We screened the FDA-approved drug library (640 compounds) and the ICCB library of known bioactives (480 compounds) largely containing compounds in clinical use with well-characterized bioactivity, bioavailability and biosafety properties. The compounds were generally screened at concentrations between 2 and 50 μM, with the exception of potent bioactive lipids, which were screened at concentrations between 0.2 and 2 μM.

We created publically accessible webpages (https://www.iai.kit.edu/askme/ChIn/) visualizing all raw screening data including soft links to relevant heterogeneous biological and chemical databases, thus providing a valuable resource for further data analysis.

Based on phenotypic observations we determined three immune modulatory categories (anti-resolution, pro- and anti-inflammatory) and empirically defined parameters for the automated classification of screened compounds. A summary of the distinct immune modulatory classes and additional observed phenotypes are reported (S1–S5 Tables). To this end, we generated a read-out, the inflammatory index, which is a measure for the degree of leukocyte infiltration at injured neuromasts. Leukocyte numbers at injured neuromasts in controls peaked at 90 min after initial copper treatment and inflammation resolved within 5 hours (S1 Fig). To identify pro- and anti-inflammatory drugs we compared the initial inflammatory index at peaking inflammation of each compound to that of the average CuSO_4_ control (100%) (S1B and S1C Fig). Anti-inflammatory drug action was defined by an initial inflammatory index ≤ 50%. Pro-inflammatory drug action was defined by an initial inflammatory index ≥ 150%. Anti-resolution drugs were characterized by inflammatory indices ≥ 50% at 5 h post-wounding, when inflammation was already resolved in controls. With these thresholds 70 anti-inflammatory hits (6%) from diverse pharmacological classes were identified (Fig 1B, S1 Table). Seven compounds were NSAIDs, confirming the validity of our screening approach, and 68 compounds (6%) were classified as pro-inflammatory (Fig 1B and S2 Table). At the tested concentrations 9 compounds (1%) prevented inflammation resolution over time (Fig 1B and S1D Fig, S3 Table) and 48 drugs (4%) induced toxic or severe adverse effects (Fig 1B and S1E Fig, S4 Table).

Hair cell viability after drug and copper treatment was assessed to identify injury-preventing drugs (S1F Fig, S5 Table), which would otherwise be automatically falsely identified as anti-inflammatory drugs. To this end, the nucleic acid stain Sytox Blue was used to reliably visualize hair cell integrity (S2 Fig). Sytox Blue is sequestered specifically in intact hair cells. Once nuclear fragmentation and hair cell extrusion from neuromasts occurs due to CuSO4-insult, the fluorescent signal vanishes. This approach identified 38 (3%) injury-preventing drugs, which were excluded from subsequent analysis (Fig 1B).

**Fig 2:**
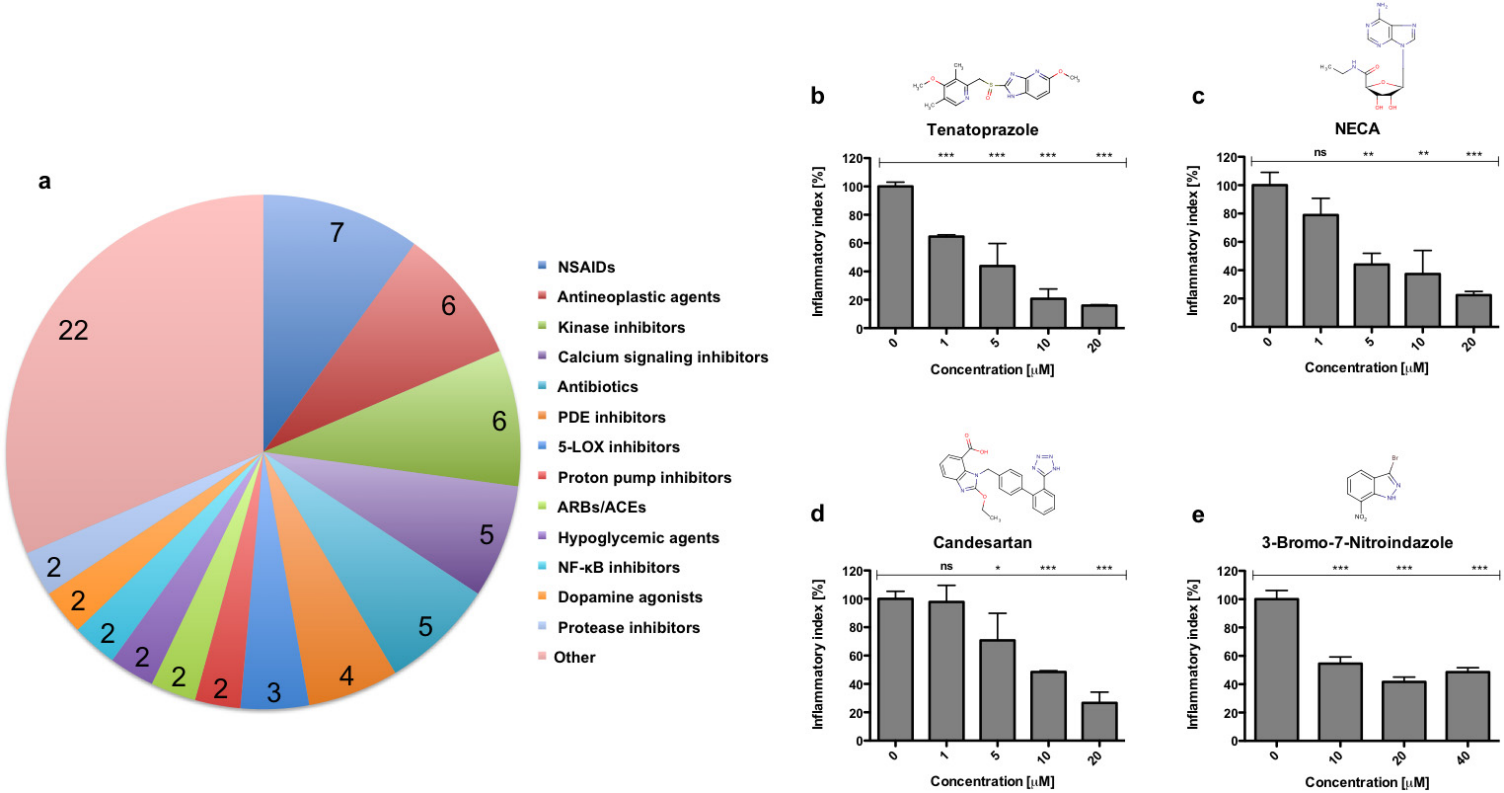
Functional classification and validation of selected anti-inflammatory hits. Anti-inflammatory candidates were classified in functional or therapeutic groups. Selected anti-inflammatory hit compounds exert dose-dependent anti-inflammatory effect on chemically induced inflammation. **(a)** Pie chart dividing the 70 anti-inflammatory hit compounds in functional or therapeutic groups containing at least 2 representatives (numbers). Groups with only one representative are summarized in “other”. (**b—e)** Dose-response behavior of selected anti-inflammatory hit compounds Tenatoprazole **(b)**, NECA **(c)**, Candesartan **(d)**, and 3-Bromo-7-nitroindazole **(e)**. Bar charts indicate means ± SEM of the initial inflammatory index (%) 90 minutes after CuSO_4_ treatment (t = 0). Three individual experiments were performed with 15 replicate larvae for each condition, respectively. Statistics were evaluated with unpaired one-sided t-tests. ns–not significant: P > 0.05, * *P* < 0.05, ** *P* < 0.01, *** *P* < 0.001.

We further focused our analyses on the anti-inflammatory category. Grouping anti-inflammatory hits by function or therapeutic indication (Fig 2A) revealed that NSAIDs (n = 7; 10%), antineoplastic agents and kinase inhibitors (n = 6; 9%), as well as antibiotics and calcium signaling inhibitors (n = 5; 7%) were the most represented drug classes.

To validate the anti-inflammatory effect of selected candidates from the primary screen we chose the following representatives of different drug classes (Fig 2B–2E): the proton pump inhibitor Tenatoprazole, the adenosine receptor agonist NECA, the Angiotensin II Type I receptor blocker Candesartan, and the nitric oxide synthase (NOS) 1 specific inhibitor 3-Bromo-7-nitroindazole. Dose-response analysis of these compounds revealed an increase in anti-inflammatory drug action with increasing inhibitor concentrations for all tested drugs. The anti-inflammatory effect of the NOS1 inhibitor suggests a pro-inflammatory role for NO in a peripheral sensory nervous system injury model. We were thus interested to further evaluate the role of NO in mediating inflammatory signals.

### NO is a pro-inflammatory signal in peripheral nervous system and epithelial tissue injuries

**Fig 3:**
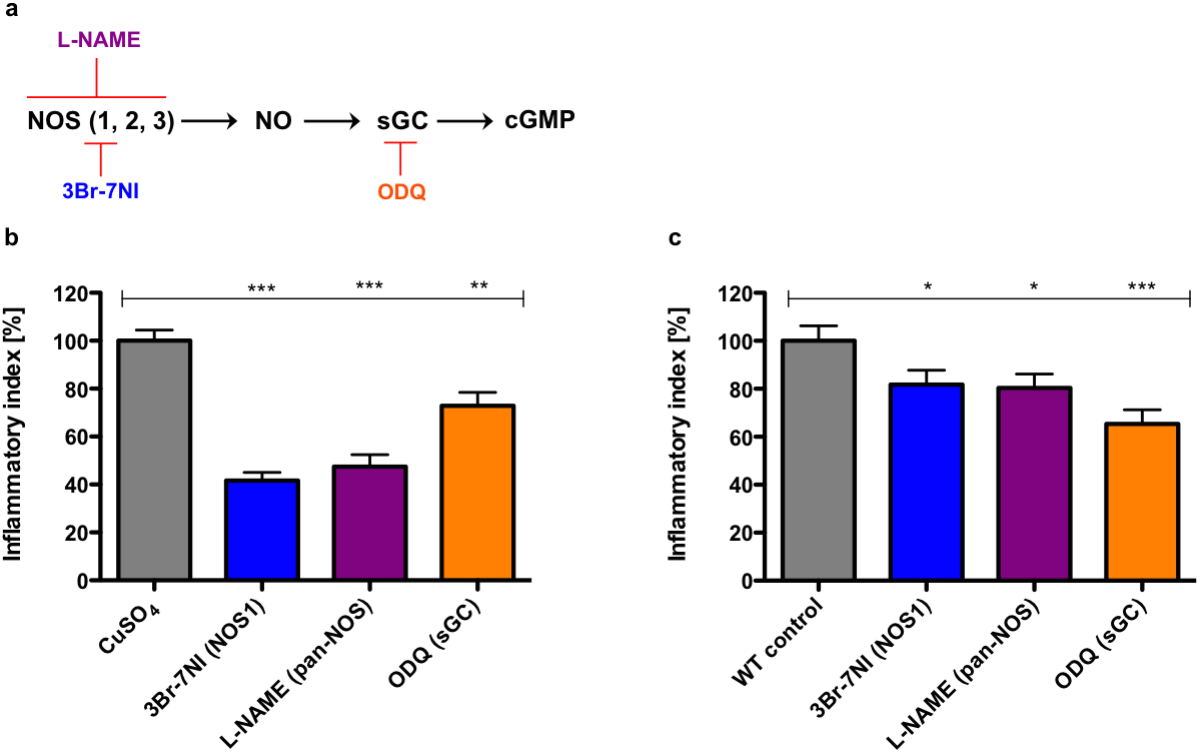
Nitric oxide is a pro-inflammatory signal upon peripheral sensory nervous system and epithelial tissue injuries. Inhibition of NOS and sGC significantly impairs leukocyte-to-wound attraction in both inflammation scenarios. **(a)** Schematic of the NO signaling pathway. Relevant chemical inhibitors acting on individual pathway components are indicated and color-coded. **(b, c)** Bar charts representing the effect of the pan-NOS inhibitor L-NAME (magenta) (ChIn: 250 μM, ventral: 1000 μM), the NOS1 inhibitor 3-Bromo-7-nitroindazole (blue) (3Br-7NI) (20 μM) and the sGC inhibitor ODQ (orange) (20 μM) on inflammation of the peripheral sensory nervous system assessed by the ChIn assay **(b)** and on epithelial inflammation assessed by mechanically induced injury of the ventral fin **(c)**. Bar charts represent means of the inflammatory index (%) ± SEM. Three individual experiments were performed with 15 replicate larvae for each condition, respectively. Statistics were evaluated with unpaired one-sided t-tests. ns–not significant: P > 0.05, * *P* < 0.05, ** *P* < 0.01, *** *P* < 0.001.

To elucidate the universality of NO relevant for attraction of leukocytes to sites of wounding, we studied the effect of different inhibitors of the NO signaling pathway in a peripheral nervous system (ChIn) and an epithelial injury setting (Fig 3). Epithelial wounds were generated by introducing an incision in the ventral fin fold and leukocyte influx to the site of wounding was measured. In vertebrates three genetically distinct isoforms of NO synthase (NOS) have been described [8]. The neuronal (nNOS, NOS1) and endothelial (eNOS, NOS3) isoforms are constitutively expressed in multiple tissues and are regulated by calcium/calmodulin, while the inducible NO synthase (iNOS, NOS2) can be transcriptionally induced especially in macrophages as a cytotoxic response mechanism [9]. Due to tissue-specific differences in the expression of distinct NOS isoforms, we tested two NOS inhibitors with differing isoform selectivity. We used the pan-NOS inhibitor L-NAME in addition to our anti-inflammatory hit candidate the NOS1 inhibitor 3-Bromo-7-Nitroindazole (3Br-7NI). To investigate the relevance of the NO-sGC-cGMP axis, we further used an inhibitor (ODQ) for the soluble guanylate cyclase. Similar to 3Br-7NI, the pan-NOS inhibitor L-NAME also produced a significant anti-inflammatory effect in the peripheral nervous system wound setting (Fig 3B). Moreover, both inhibitors also resulted in a significant reduction of leukocyte infiltration in the epithelial wound setting (Fig 3C). Therefore, NO is produced as a tissue-independent immediate pro-inflammatory wound signal. Furthermore, inhibition of sGC with ODQ resulted in a significant anti-inflammatory effect in both wounding scenarios implicating the NO-sGC-cGMP axis in leukocyte-to-wound attraction.

### Nos2b mediates NO production in both inflammation scenarios

In order to identify the relevant zebrafish Nos isoform in inflammation, we applied genetic loss-of-function by morpholino knockdown of zebrafish *nos1* (nNOS), *nos2a* (iNOS) and *nos2b* as well as *gucy1a3* (sGC) to attenuate NO signaling (S3 Fig). The zebrafish genome includes one *nos1* and two *nos2* genes but no distinct *nos3* gene. Zebrafish *nos2a* expression is inducible *in vitro* and *in vivo* by lipopolysaccharide [10]. Thus, the zebrafish Nos2a behaves as the mammalian inducible NOS (iNOS) isoform. The *nos2b* gene is constitutively expressed during development and in most adult zebrafish tissues. Along with an N-terminal myristoylation consensus sequence, this suggests functional homology with mammalian NOS3 (eNOS) [10].

**Fig 4:**
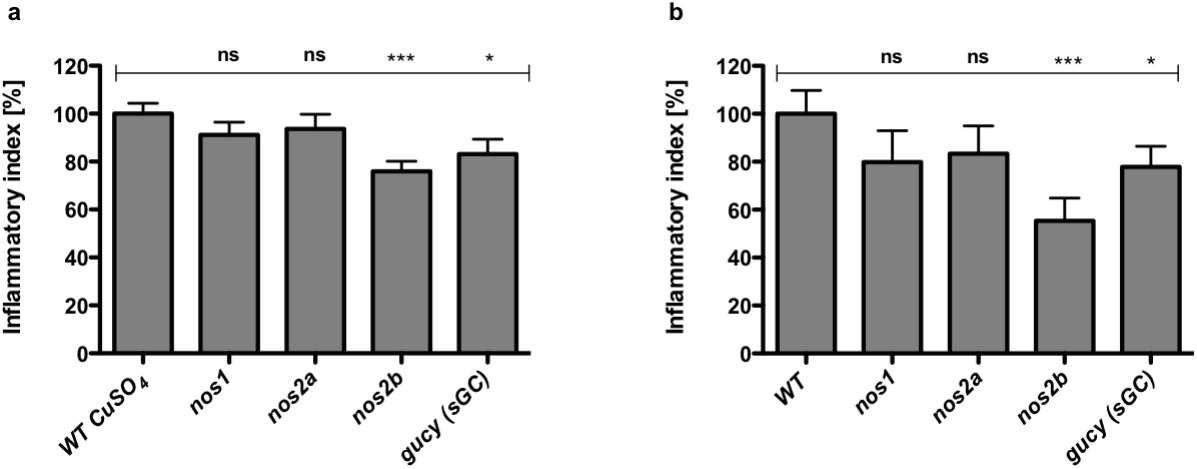
Genetic knockdown of zebrafish *nos2b* and sGC (*gucy*) but not *nos1* and *nos2a* impair the inflammatory response. Assessment of the role of the 3 zebrafish Nos isoforms and sGC on peripheral sensory nervous system **(a)** and epithelial **(b)** inflammation upon genetic knockdown. **(a)** *nos2b* and *gucy* knockdown significantly reduce initial inflammation at injured neuromasts as compared to wildtype (WT) larvae. **(b)** Knockdown of *nos2b* and *gucy* significantly reduce the number of neutrophils recruited to ventral fin wounds. Bar charts represent means of the initial inflammatory index (%) ± SEM 90 minutes after CuSO_4_ treatment **(a)** and 1 h after mechanical injury of the ventral fin **(b)**. Three individual experiments were performed with 15 replicate morphant larvae for each condition, respectively. Statistics were evaluated with unpaired one-sided t-tests. ns–not significant: P > 0.05, * *P* < 0.05, ** *P* < 0.01, *** *P* < 0.001.

Anti-inflammatory activity of 3Br-7NI in both wounding scenarios suggested involvement of zebrafish Nos1 in both peripheral sensory nervous system and epithelial inflammation. Genetic knockdown experiments of all zebrafish *nos* isoforms however only confirmed a significant reduction of leukocyte-to-wound attraction upon knockdown of *nos2b* in both wounding scenarios (Fig 4).

The soluble guanylate cyclase of mammals is an obligate heterodimer, consisting of one α and one β subunit. Most tissues express a heterodimer of α_1_β_1_ [11]_._ To knockdown sGC activity we therefore targeted the zebrafish α_1_ subunit (*gucy1a3*). A significant reduction of leukocyte infiltration upon sGC knockdown was observed in both inflammatory settings (Fig 4).

### Nitric oxide production results in protein S-nitrosylation during inflammation

**Fig 5:**
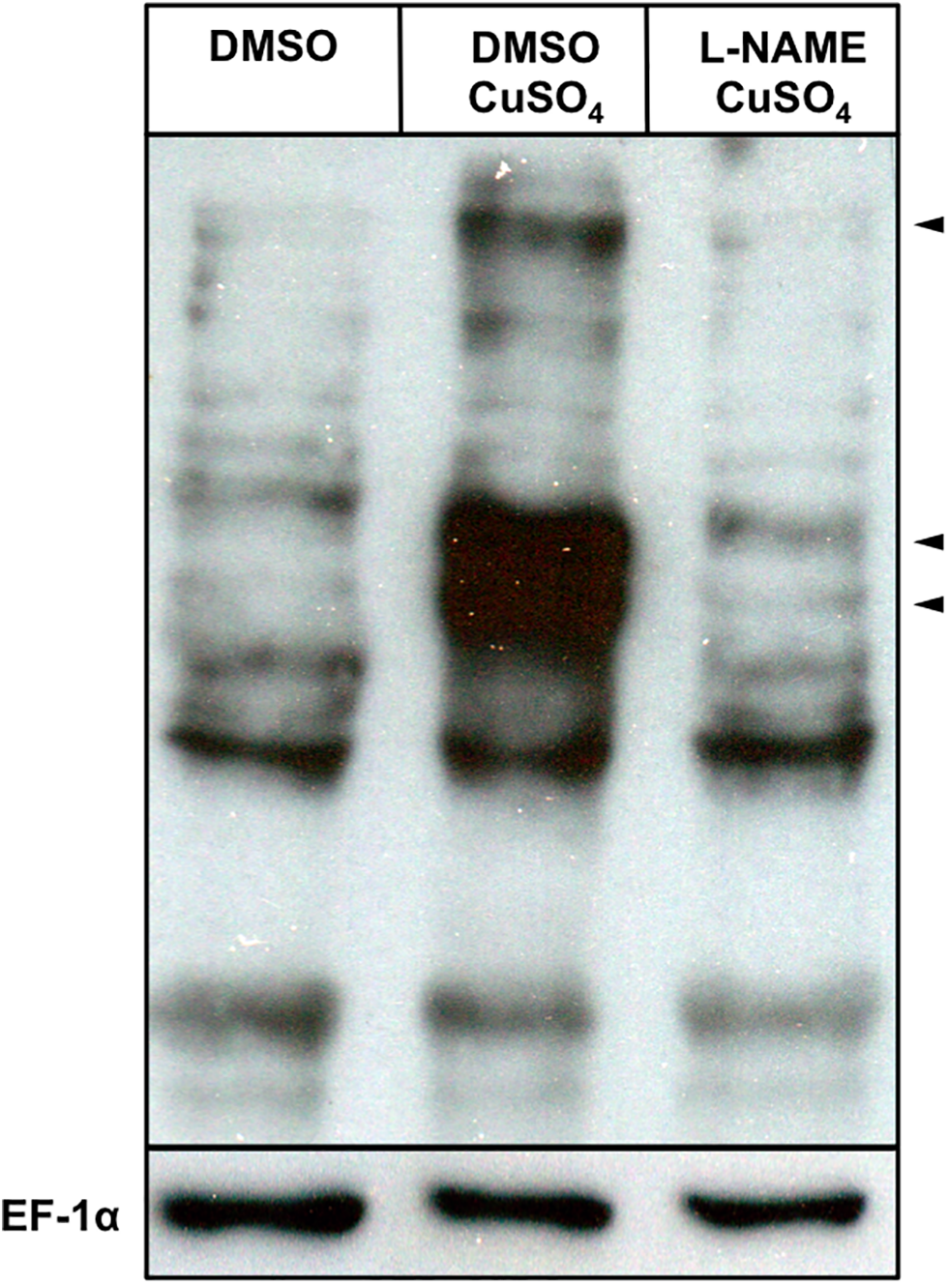
Inflammation induces S-nitrosylation in zebrafish larvae. Changes in total protein S-nitrosylation during inflammation was assessed with the modified biotin switch technique and detected by Western Blot. S-nitrosylated proteins in samples of control (lane 1) larvae and larvae with chemically induced inflammation (lane 2 and 3) were detected by anti-TMT immunoblotting. Chemically induced inflammation significantly induces S-nitrosylation, marked by additional strong anti-TMT immunoblotting signals (arrowheads), in protein extracts of CuSO_4_ treated larvae. Inhibition of Nos with 250 μM L-NAME prior to CuSO_4_ treatment prevents changes in protein S-nitrosylation. EF–1α was used as loading control.

Alternatively, NO can exert effects by redox-based posttranslational protein modification (S-nitrosylation). In order to investigate whether protein S-nitrosylation is a viable regulatory mechanism during inflammatory processes, we determined changes in total protein S-nitrosylation during CuSO_4_ induced inflammation using the modified biotin-switch technique. We observed differences in the degree of total protein S-nitrosylation between DMSO control and CuSO_4_ treated groups (Fig 5). Total protein S-nitrosylation, marked by the appearance of additional strong anti-TMT immunoblotting signals significantly increased upon CuSO_4_ treatment. Nos inhibition with 250 μM L-NAME prior to CuSO_4_ treatment prevented injury induced protein S-nitrosylation, suggesting a contribution of sGC-independent NO-mediated mechanisms modulating the wound inflammatory response.

In recent years several phenotypic screens for modulators of behavior [12], modulators of glucocorticoid signaling activity [13], hematopoietic stem cell formation [14], and others [15–19] were successfully conducted in zebrafish. The translational relevance of drug discovery in zebrafish is emphasized by the fact that several compounds identified in zebrafish screens were successfully evaluated in preclinical trials [20,21] and even proceeded to phase 1b clinical trials [5]. Whole organismal screens offer the unique advantage of studying interactions of compounds with a physiological process of choice in its entire complexity. A multifaceted physiological process such as the inflammatory response can be phenotypically assessed in zebrafish in the presence of a fully functional innate immune system. The migration of leukocytes to sites of injuries upon compound treatment allows detailed examination of the molecular players involved in surveillance of tissue homeostasis, sensation and mediation of inflammatory signals as well as directed cell migration during an inflammatory response.

Thus far, three chemical-genetic screens have been conducted in zebrafish aiming at the identification of immune modulatory compounds [22–24]. In these screens compounds were manually assayed for the ability to suppress the recruitment of neutrophils to tail fin injuries. In comparison, the ChIn assay–based screening approach applied here offers the unique advantage of testing for immune-modulatory activities *in vivo* and in real time. Therefore, the described setup allows assessment of compounds modulating inflammation initiation and resolution as well as revealing adverse and toxic drug effects. Importantly, chemical wounding renders manual manipulation of larvae obsolete, thus permitting automation and screening in a high-throughput format. To our knowledge, this study represents one of the first fully automated *in vivo* drug-repositioning screen performed in 384-well plate format [18,19]. One drawback of chemically induced inflammation however is the possibility that compounds may interfere with the hair cell damaging agent CuSO_4_, thereby preventing neuromast damage. We therefore identified injury-preventing drugs through assessment of hair cell viability after drug and copper treatment and found 38 compounds (3%) in this category. The mode of action underlying the hair cell protective effect was not further investigated. It is likely that many of the injury preventing drugs have chemical properties capable of chelating Cu^2+^-atoms through the formation of two or more coordinate bonds, thereby attenuating the detrimental CuSO4 effects. Similarly, general otoprotective effects may also reduce the cell damaging activity of CuSO_4_. However, the relatively minor proportion of drugs preventing hair cell damage upon CuSO4 treatment can be probed for true immune modulatory activity in a low-throughput copper-independent mechanical wounding assay.

We here focused on the anti-inflammatory category of compound hits and defined anti-inflammatory drug activity by a significant reduction in neutrophil numbers at damaged neuromasts in analogy to the IC50 value. This threshold resulted in 70 anti-inflammatory modulators representing 6% of the screened drugs. The 70 compounds represent 13 different drug classes according to function or therapeutic indication with two or more compound hits. In addition, 22 of these compounds represent individual therapeutic or functional classes. The largest pharmacological group within the anti-inflammatory category are NSAIDs, confirming the validity of our screening approach. At the chosen screening concentration and threshold for anti-inflammatory drug activity, 19% of all NSAIDs within the screened libraries were detected. This observation confirms that NSAIDs are generally not very potent anti-inflammatories. Other pharmacological classes, each containing 5 or more representatives with anti-inflammatory properties are antineoplastic agents and kinase inhibitors (n = 6; 9%). 7% of all hits (n = 5) were inhibitors of calcium signaling and antibiotics, respectively. Calcium is one of the most immediate messengers upon mechanical and laser induced wounding in neuronal and epithelial tissues initiating the wound inflammatory response in different model organisms [25–27]. In these studies, initial wound-derived calcium signaling from extra- and intracellular stores proved indispensible for immune cell recruitment to sites of injuries and for subsequent regenerative responses. We identified different calcium channel inhibitors specific for inhibition of calcium influx or release from both extra- and intracellular sources to significantly suppress leukocyte-to-wound attraction. These results suggest a critical role of calcium from both extra- and intracellular sources also for efficient mounting of the wound inflammatory response in our chemically induced peripheral sensory nervous system injury model.

For hit validation we positively validated representatives of four compound classes in dose-response-behavior analysis using the chemically induced inflammation assay.

Angiotensin II Type 1 receptor blockers (ARBs) such as Candesartan are reportedly effective in hypertension related vascular inflammation in peripheral organs. Our observations with Candesartan confirm the hypothesized immune modulatory activity of ARBs beyond hypertension [28].

Proton pump inhibitors (PPIs) such as Tenatoprazole are used in the treatment of gastro-oesophageal reflux disease to inhibit the H^+^/K^+^ ATPase of gastric parietal cells. Recently, a number of mechanisms were discussed based on *in vitro* data, whereby PPIs may exert anti-inflammatory effects unrelated to the inhibition of gastric acid production [29]. In accordance, we here report an *in vivo* inhibitory effect of PPIs on leukocyte recruitment to sites of peripheral nervous system injuries.

NECA is a selective agonist of adenosine A2A receptors expressed on leukocytes [30]. Signaling via A_2A_ receptors suppresses inflammatory and immune responses by reducing the production of many pro-inflammatory cytokines and inhibition of adhesion molecule expression [31].

3-Bromo-7-nitroindazole is described as a NOS1 inhibitor. Measurements in experimental animal models with spinal cord injuries revealed that NO accumulates and nitric oxide synthase (NOS1) activity increases in injured tissues [32]. In addition, a distinct temporal pattern of nitric oxide production with modulation of NO concentrations marked by an immediate increase, a decrease between 1 and 12 h after injury and a second wave of NO production between 24 h and 3 days after injury was described. The later wave of NO production is attributed to inducible nitric oxide synthase (NOS2) expression in damaged tissues starting approximately 2 h after injury and steadily increasing thereafter [32]. Despite the well documented NO accumulation in injured tissues, the actual role of NO upon tissue injury is controversially discussed. Some reports argue for a protective, others for a detrimental role of NO with respect to neurotoxic and neuroprotective effects after traumatic tissue injury.

Although the function of NO upon neuronal injuries is certainly complex, our *in vivo* data suggest that NO modulates leukocyte attraction to peripheral sensory nervous system injuries indicating that modulation of NO signaling might be therapeutically beneficial for patients with acute and chronic inflammatory conditions. Dibaj *et al*. reported a role for NO in the recruitment of microglia to lesions of spinal cord white matter in mice [33]. Superfusion with NOS inhibitors prior to spinal cord white matter tissue damage inhibited microglial extensions towards the lesion. Taken together with the results presented here, these studies indicate that NO attracts leukocytes specifically to neuronal and peripheral nervous system injuries. Our observation that inhibition of Nos also reduced neutrophil recruitment to mechanically inflicted epithelial wounds in the zebrafish ventral fin extends the role of NO to a conserved pro-inflammatory one in both neuronal and epithelial injury settings.

Pharmacological inhibition of sGC, the only known receptor for NO, significantly reduced attraction of leukocytes to sites of peripheral sensory nervous system and epithelial injuries. It is therefore conceivable that sGC may act as a receptor on leukocytes, perceiving directional cues from NO, regulating chemotaxis of leukocytes. This notion is also in line with findings by Duan and Dibaj *et al*. who reported that sGC inhibition affected directional movement of microglia, resulting in reduced microglia accumulation at leech nerve cord lesions and spinal cord white matter injuries in mice [33,34]. Our data suggest that NO may also contribute to the inflammatory response in an sGC-independent manner via protein S-nitrosylation. sGC-mediated NO effects may primarily target immune cells, while S-nitrosylation governs NO responses in cells at and around the site of injury affecting the production of secondary pro-inflammatory mediators. For example, S-nitrosylation activates cytosolic phospholipase A_2_α, which is the rate-limiting key enzyme cleaving arachidonic acid from membrane phospholipids for eicosanoid production including prostaglandin E_2_ [35]. Furthermore, S-nitrosylation activates cyclooxygenase–2 and enhances its catalytic activity. Consequently, sGC-independent NO action results in the production of pro-inflammatory lipid mediators. Other known NO-regulated targets are matrix metalloproteinases, most importantly MMP–9, which are involved in remodeling of extracellular matrix [36]. MMP–9 activation by protein S-nitrosylation may thus facilitate leukocyte tissue transmigration.

There is ample evidence of an intricate interplay between NO and calcium signaling. NO production by constitutive nitric oxide synthases (NOS1 and NOS3) is regulated by intracellular calcium concentrations [8]. Simultaneously, NO is implicated in affecting calcium signaling. For instance, NO was suggested as a paracrine messenger promoting intracellular calcium wave propagation [37]. Additionally, NO has implications in regulating extra- and intracellular calcium flux through protein S-nitrosylation via TRP channels [38] and IP_3_ receptors [39], respectively. Accordingly, we found several calcium channel inhibitors including the IP_3_ receptor inhibitor 2-APB and the TRP channel modulator 6-gingerol with significant anti-inflammatory properties. NO may thus act as a feed-forward signal to promote and/or sustain injury-induced calcium signaling. It will be intriguing to decipher actual targets of S-nitrosylation in an inflammatory context.

Through genetic inhibition we identified the relevant zebrafish Nos isoform critical for efficient mounting of an inflammatory response. Genetic inhibition of all Nos isoforms revealed that only zebrafish *nos2b* significantly reduced leukocyte-to-wound attraction. Interestingly, heterocyclic compounds such as 7-Nitroindazole (7-NI) were reported to have greater selectivity towards NOS1 *in vivo*, while exhibiting equipotent efficiency for all NOS isoforms when examined against isolated enzyme preparations [40]. While our pharmacological data using the 7-NI derivative 3Br-7NI would therefore suggest zebrafish Nos1 as the relevant isoform, the genetic data clearly identifies zebrafish Nos2b in the described inflammatory context. Thus 3Br-7NI efficiently inhibits Nos2b in an *in vivo* zebrafish inflammation model.

This work described here employs a fully automated *in vivo* phenotypic screening approach in zebrafish larvae providing a comprehensive data set for systematic immune modulatory drug discovery in the context of an intact innate immune system. Further investigation of nitric oxide signaling upon the anti-inflammatory activity of a nitric oxide synthase inhibitor substantiates the role of NO as an important tissue-independent pro-inflammatory mediator critical for the attraction of leukocytes to wounds. Further, this study provides new mechanistic insights into nitric oxide mediated regulation of inflammatory processes.

## Methods

### Aquaculture and transgenic fish lines

Zebrafish husbandry and experimental procedures were performed in accordance with the German animal protection standards and were approved by the Regierungspräsidium Karlsruhe, Germany (General license for fish maintenance and breeding: Az.: 35–9185.64). All embryos were collected by natural spawning, and raised at 28°C in E3 medium (see S6 Table for transgenic lines used). All animals subjected to experimentation were anesthetized in MS–222 (Sigma-Aldrich A5040).

### Chemical Libraries and pharmacological perturbations

The FDA-approved drug library (BML–2841 v.1.4) and the ICCB library of known bioactives (BML–2840 v.2.1) (Enzo Life Sciences) were screened at concentrations ranging from 0.2–50 μM. Anti-inflammatory candidate compounds were purchased at Santa Cruz Biotechnology and tested at drug concentrations ranging from 1–20 μM. Drugs affecting NO signaling such as 3-Bromo-7-nitroindazole (NOS1), 1H-[1,2,4]Oxadiazolo[4,3-a]quinoxalin-1-one (ODQ) (sGC), and Nω-Nitro-L-arginine methyl ester (L-NAME) hydrochloride (pan-NOS) were used at concentrations ranging from 10–50 μM and 250–1000 μM, respectively.

### Automated chemically induced inflammation assay (ChIn_a_)

The ChIn_a_ assay was performed as previously described [7]. Images were acquired on an inverted automated Olympus Scan**⌃**R microscope 90 minutes after initial copper treatment. Each well was imaged once per hour for six hours in the channels brightfield, Cy3 and GFP in 4 focal planes (50 μm distance) using a 4x objective (N.A. = 0.13).

### Quantification of inflammation

Image processing and quantification of inflammation was accomplished with custom developed software scripts based on LabView and MATLAB [7]. In brief, extended focus RGB overlay images from the channels GFP, brightfield and Cy3 were generated. Green fluorescent neuromasts were detected using a pattern recognition tool. As primary read-out we scoring the percent area occupied by leukocytes (*Paol*) in an empirically defined area of interest around injured neuromasts.

### Screening statistics

Each 384-well plate contained 320 compounds and 32 positive (CuSO_4_) and negative controls (DMSO), respectively. The *Paol* of the 32 control replicates was averaged and standard deviation was calculated. Only data points within 2 standard deviations were included. The average *Paol* of DMSO was set to 0 and the averaged *Paol* for the CuSO_4_ was set to 1. After normalization each compound’s *Paol* was linearly interpolated or extrapolated to the respective controls on the experimental plate. Normalized raw data from 15 replicate experiments were averaged, resulting in the inflammatory index. A monotonic exponential nonlinear regression fitting towards the initial inflammation was performed $e^{\left(a_{0}+a_{1}t\right)}$. A_0_ is a measure for the initial response at t = 0, a_1_ is related to the slope of magnitude over time. The initial inflammatory index (%) displayed in bar charts was obtained by solving the nonlinear regression ($e^{\left(a_{0}+a_{1}t\right)}$) for t = 0.

### Immune modulatory categories

Anti-inflammatory drug action was defined by an inflammatory index ≤ 50% (a_0_ ≤ -0.68) 90 minutes after initial copper treatment (t = 0). The threshold for pro-inflammatory drugs was set to an inflammatory index ≥ 150% (a_0_ ≥ 0.41) at t = 0. The threshold parameters for anti-resolution drugs were a_0_ ≥ -0.21 (inflammatory index ≥ 80% at t = 0) and a_1_ ≥ -0.12. Compounds were considered as “normal” with the following parameters: -0.68 < a_0_ < 0.41 and a_1_ < -0.12.

### Screening results

All screening data is accessible at https://www.iai.kit.edu/askme/ChIn/

### Manual chemically induced inflammation assay (ChIn_m_)

The manual ChIn assay was performed with morphant larvae. The manual ChIn assay was carried out analogous to the automated ChIn assay, with minor variations. All treatments were performed in 6-well plates in a volume of 3 ml. The inflammatory response was quantified by manual counting of leukocytes within the horizontal myoseptum [41]. The inflammatory index was obtained by normalization of experimental conditions to the average cell count of the CuSO_4_ control.

### Ventral fin assay

Epithelial inflammation was assessed in ventral fins of Tg(lyz:DsRED2)nz50 larvae upon nicking the ventral fin fold of anesthetized larvae with a sterile scalpel. Larvae were left to recover for 1 h in presence or absence of drug prior to microscopic analysis. Fluorescent leukocytes were counted in an area of interest around the wound. The inflammatory index was obtained by normalization of experimental conditions to the average cell count of the positive control.

### Identification of compounds preventing CuSO_4_ induced neuromast damage

Hair cell damage was assessed using the nuclear acid stain Sytox Blue (Molecular Probes S34857). Larvae were incubated for 2 h in E3 supplemented with DMSO (1%) and Sytox Blue (5 μM final concentration) and washed two times with E3 containing 1% DMSO and MS–222 prior to the automated ChIn assay procedure. Brightfield and DAPI images were acquired 90 minutes after initial CuSO4 treatment to assess hair cell integrity.

### Morpholino knockdown

Morpholino oligonucleotides were obtained from Gene Tools and injected at non-toxic concentrations (MO*nos1*: 0.08 mM, MO*nos2a*: 0.4 mM, MO*nos2b*: 0.2 mM, MO*gucy1a3*: 0.15 mM) into one-cell stage zebrafish embryos. Gene knockdown was confirmed by RT-PCR (S3 Fig; S6 Table).

### Detection of S-nitrosylation

S-nitrosylation was analyzed in larval extracts using the S-nitrosylation Western Blot kit (Thermo Scientific 90105) according to the manufacturer’s protocol with minor variations mentioned below. Larvae were treated with L-NAME (250 μM) or DMSO (1%) for 1 h, followed by 10 μM CuSO_4_ treatment for 45 min at 28°C in the dark and washed 3 times with PBS supplemented with MS–222. Proteins were extracted in HENS buffer. 60 μg of protein was used per labeling reaction. MMTS was used at 200 μM and blocking was performed at 50°C with constant shaking and 10 μg of protein was loaded per lane.

### Statistical Analysis

Statistical analyses were performed using Prism 5.0 (GraphPad). Drug effects were assessed by comparing inflammatory indices of drug versus control using unpaired, one-tailed t-tests. All data presented in bar charts show means ± SEM.

## Supporting Information

S1 FigInflammatory kinetics of the major immune modulatory categories, injury preventing compounds and toxic compounds.Kinetics of the inflammatory response of 1120 compounds from the FDA- approved library and the ICCB library of known bioactives. Individual graphs display the inflammatory index over time based on non-linear regression fitting of original data using $e^{\left(a_{0}+a_{1}t\right)}$ for the different categories. Red dots in each graph represent the inflammatory index of the averaged CuSO4 control. Black curves represent individual compounds. **(a)** Compounds with normal inflammatory response. **(b)** Drugs with anti-inflammatory activity. **(c)** Pro- inflammatory compounds. **(d)** Anti-resolution compounds. **(e)** Toxic compounds and compounds with severe adverse effects. **(f)** Injury preventing compounds. The majority of compounds in this category mimic anti- inflammatory drug activity.(PDF)

S2 Fig Identification of injury preventing compounds with Sytox Blue. Images show 3 dpf wildtype larvae stained with Sytox Blue **(a)** DMSO control. Sytox Blue is sequestered to intact hair cells (indicated with white arrowheads). **(b)** CuSO4 control. Sytox Blue fluorescence vanishes upon nuclear fragmentation due to CuSO4 treatment. (**c)** Exemplary injury preventing compound. Intact hair cells are marked with white arrowheads.
(PDF)

S3 Fig Morpholino knockdown of three zebrafish *nos* isoforms and *gucy1a3*. Knockdown efficacy was assessed by semi-quantitative measurements of altered or reduced transcript levels via RT-PCR. RT-PCR was performed on batches of 15 pooled splice site morpholino (MO) injected (+) and control (-) larvae, respectively. Injection of MO*nos1* (0.08 mM) splice site MO results in an additional shorter PCR product. Injection of MO*nos2a* (0.4 mM) significantly reduces *nos2a* transcript levels. Successful knockdown of *nos2b* by injection of the splice blocking MO (0.2 mM) is evident by generation of alternate PCR products. Efficient knockdown of *gucy1a3* in (0.15 mM) MO injected larvae results in generation of transcripts shorter or longer than wildtype, respectively.
(PDF)

S1 Table (Related to Fig 1) Anti-inflammatory compounds. (PDF)

S2 Table (Related to Fig 1) Pro-inflammatory compounds. (PDF)

S3 Table (Related to Fig 1) Anti-resolution compounds. (PDF)

S4 Table (Related to Fig 1) Toxic compounds and compounds with adverse effects. (PDF)

S5 Table (Related to Fig 1) Injury preventing compounds. (PDF)

S6 Table Transgenic zebrafish lines, oligonucleotide and morpholino sequences. (PDF)
