# Supplementary material for: A Zebrafish Drug-Repurposing Screen Reveals sGC-Dependent and sGC-Independent Pro-Inflammatory Activities of Nitric Oxide
Source: PLoS One. 2015 Oct 7;10(10):e0137286. doi: 10.1371/journal.pone.0137286 (PMC4596872; doi:10.1371/journal.pone.0137286)
Supplement: S1 Fig — Kinetics of the inflammatory response of 1120 compounds from the FDA- approved library and the ICCB library of known bioactives. Individual graphs display the inflammatory index over time based on non-linear regression fitting of original data using e(a0+a1t) for the different categories. Red dots in each graph represent the inflammatory index of the averaged CuSO4 control. Black curves represent individual compounds. (a) Compounds with normal inflammatory response. (b) Drugs with anti-inflammatory activity. (c) Pro- inflammatory compounds. (d) Anti-resolution compounds. (e) Toxic compounds and compounds with severe adverse effects. (f) Injury preventing compounds. The majority of compounds in this category mimic anti- inflammatory drug activity. (PDF) [file pone.0137286.s001.pdf]

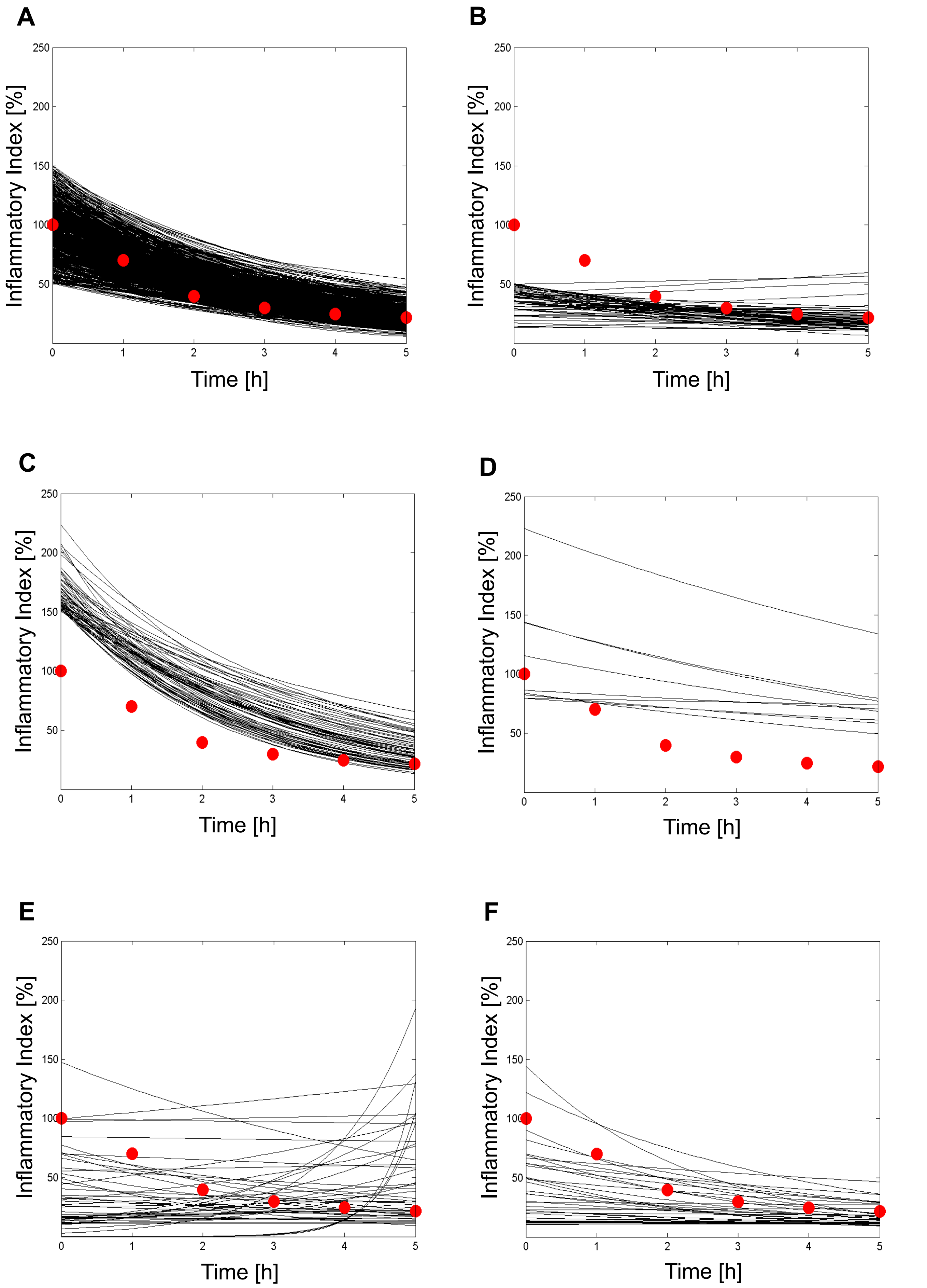
A
B
C
D
E
F
Inflammatory Index [%]
Time [h]
250
200
150
100
50
0
1
2
3
4
5

S1 Fig. Inflammatory kinetics of the major immune modulatory categories, injury preventing compounds and toxic compounds.

Kinetics of the inflammatory response of 1120 compounds from the FDA-approved library and the ICCB library of known bioactives. Individual graphs display the inflammatory index over time based on non-linear regression fitting of original data using $e^{(a0+a1t)}$ for the different categories. Red dots in each graph represent the inflammatory index of the averaged $CuSO_4$ control. Black curves represent individual compounds. **(A)** Compounds with normal inflammatory response. **(B)** Drugs with anti-inflammatory activity. **(C)** Pro-inflammatory compounds. **(D)** Anti-resolution compounds. **(E)** Toxic compounds and compounds with severe adverse effects. **(F)** Injury preventing compounds. The majority of compounds in this category mimic anti-inflammatory drug activity.
