# Supplementary material for: A Zebrafish Drug-Repurposing Screen Reveals sGC-Dependent and sGC-Independent Pro-Inflammatory Activities of Nitric Oxide
Source: PLoS One. 2015 Oct 7;10(10):e0137286. doi: 10.1371/journal.pone.0137286 (PMC4596872; doi:10.1371/journal.pone.0137286)
Supplement: S2 Fig — Images show 3 dpf wildtype larvae stained with Sytox Blue (a) DMSO control. Sytox Blue is sequestered to intact hair cells (indicated with white arrowheads). (b) CuSO4 control. Sytox Blue fluorescence vanishes upon nuclear fragmentation due to CuSO4 treatment. (c) Exemplary injury preventing compound. Intact hair cells are marked with white arrowheads. (PDF) [file pone.0137286.s002.pdf]

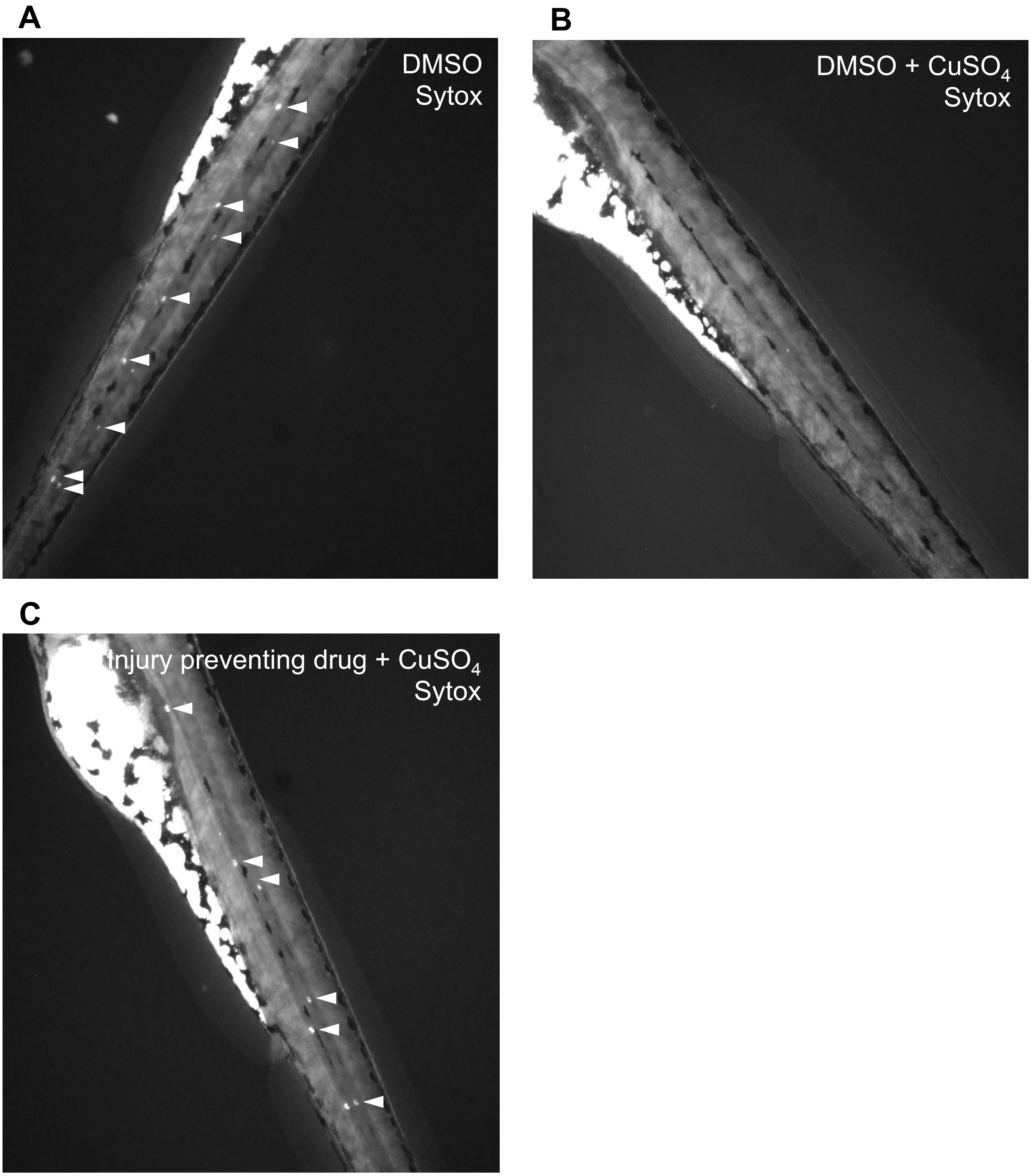

S2 Fig. Identification of injury preventing compounds with Sytox Blue.

Images show 3 dpf wildtype larvae stained with Sytox Blue **(A)** DMSO control. Sytox Blue is sequestered to intact hair cells (indicated with white arrowheads). **(B)** $CuSO_4$ control. Sytox Blue fluorescence vanishes upon nuclear fragmentation due to $CuSO_4$ treatment. **(C)** Exemplary injury preventing compound. Intact hair cells are marked with white arrowheads.
