# Supplementary material for: A Zebrafish Drug-Repurposing Screen Reveals sGC-Dependent and sGC-Independent Pro-Inflammatory Activities of Nitric Oxide
Source: PLoS One. 2015 Oct 7;10(10):e0137286. doi: 10.1371/journal.pone.0137286 (PMC4596872; doi:10.1371/journal.pone.0137286)
Supplement: S3 Fig — Knockdown efficacy was assessed by semi-quantitative measurements of altered or reduced transcript levels via RT-PCR. RT-PCR was performed on batches of 15 pooled splice site morpholino (MO) injected (+) and control (-) larvae, respectively. Injection of MOnos1 (0.08 mM) splice site MO results in an additional shorter PCR product. Injection of MOnos2a (0.4 mM) significantly reduces nos2a transcript levels. Successful knockdown of nos2b by injection of the splice blocking MO (0.2 mM) is evident by generation of alternate PCR products. Efficient knockdown of gucy1a3 in (0.15 mM) MO injected larvae results in generation of transcripts shorter or longer than wildtype, respectively. (PDF) [file pone.0137286.s003.pdf]

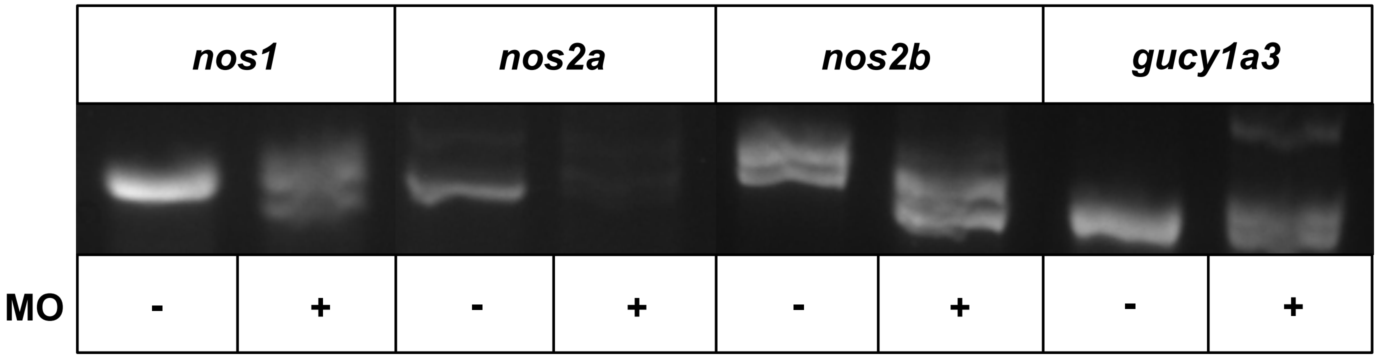

S3 Fig. Morpholino knockdown of three zebrafish *nos* isoforms and *gucy1a3.*

Knockdown efficacy was assessed by semi-quantitative measurements of altered or reduced transcript levels via RT-PCR. RT-PCR was performed on batches of 15 pooled splice site morpholino (MO) injected (+) and control (-) larvae, respectively. Injection of *nos1* (0.08 mM) splice site MO results in an additional shorter PCR product. Injection of MO *nos2a* (0.4 mM) significantly reduces *nos2a* transcript levels. Successful knockdown of *nos2b* by injection of the splice blocking MO (0.2 mM) is evident by generation of alternate PCR products. Efficient knockdown of *gucy1a3* in (0.15 mM) MO injected larvae results in generation of transcripts shorter or longer than wildtype, respectively.
