# Supplementary material for: A Zebrafish Drug-Repurposing Screen Reveals sGC-Dependent and sGC-Independent Pro-Inflammatory Activities of Nitric Oxide
Source: PLoS One. 2015 Oct 7;10(10):e0137286. doi: 10.1371/journal.pone.0137286 (PMC4596872; doi:10.1371/journal.pone.0137286)
Supplement: S1 Table — (PDF) [file pone.0137286.s004.pdf]

S1 Table (Related to Fig 1) Anti-inflammatory compounds

Compound name	Description	Screening concentration [µM]
CAPE	Inhibitors: Antioxidant/ NFkappa B inhibitor	29.31
Tenatoprazole	Proton pump inhibitor	9.63
2-APB	Inhibitors: IP3 receptor blocker	37.02
HBDDE	Kinase inhibitors: PKC inhibitor	24.63
LY-294002	Lipid biosynthesis: PI-3-Kinase inhibitor	27.12
Thapsigargin	Ion channel ligands: Intracellular calcium	12.8
Amoxicillin	Antibiotic	9.13
2,5-Ditertbutylhydroquinone	Ion channel ligands: ER Ca++ ATPase inhibitor	37.49
tyrphostin AG-126	Kinase inhibitors: tyrosine kinase inhibitor	38.72
Lavendustin A	Kinase inhibitors: Tyrosine kinase inhibitor EGF-R)	21.85
HA14-1	Inhibitors: Bcl-2 ligand induces apoptosis	20.36
Triptolide	Inhibitors: inhibits NFkappaB transcriptional activation	23.12
Troglitazone	Hypoglycemic agent	7.56
Pantoprazole	Proton pump inhibitor	8.7
Tolazamide	Ion channel ligands: Potassium channels	26.76
Cilastatin	Protease inhibitor	9.31
Candesartan	Angiotensin II Type 1 Receptor Blocker	7.57
C-PAF	Bioactive lipids: PAF receptor agonist	2
MCI-186	Inhibitors: antioxidant, cytoprotectant	47.84
Loratadine	Histamine H1 antagonist	8.72
Telmisartan	Angiotensin II Type 1 Receptor antagonist; Angiotensin-Converting Enzyme inhibitor	6.48
Diclofenac	NSAID	11.3
Ceftazidime	Anti-bacterial agent	6.1
Prothionamide	antiinfective agent; antibacterial agent	18.51
Flufenamic acid	NSAID	11.86
Diphenyleneiodonium	Inhibitors: flavoprotein inhibitor	29.86
Disulfiram	Alcohol deterrent	11.26
Progesterone	Steroid hormone	10.61
Ikarugamycin	Inhibitors: inhibits clathrin coated pit mediated endocytosis	17.34
BW-B 70C	Lipid biosynthesis: 5 lipoxygenase inhibitor	26.35
Meloxicam	NSAID	9.5

Nifedipine	Ion channel ligands: Calcium channels	24.06
Bromo-7-nitroindazole	Inhibitors: NO synthase inhibitor	34.44
Goserelin	Antineoplastic agent	2.63
Zileuton	NSAID	14.12
Spironolactone	Natriuretic Agent	8.01
Sildenafil	PDE-5 inhibitor	7.03
(R)-(-)-Apomorphine·HCl	Dopamine agonist; Antiparkinson drug	11
Vinpocetine	Inhibitors: phosphodiesterase (PDE1) inhibitor	23.78
Tenoxicam	NSAID	9.89
Mefenamic acid	NSAID	13.83
Methimazole	Antithyroid agent	29.24
6-Formylindolo [3,2-B] carbazole	Bioactive lipids: AHR agonist	2
N-Phenylanthranilic acid	Ion channel ligands: Misc. channels	39.09
U-0126	Kinase inhibitors: MEK inhibitor	21.9
H-89	Kinase inhibitors: PKA inhibitor	18.67
Bexarotene	anticarcinogenic agent	9.57
Splitomycin	Inhibitors: sir2p inhibitor	42.05
6,7-ADTN	CNS receptor ligands: Dopamine agonist	46.5
6-Gingerol	Ion channel ligands: Intracellular calcium	28.31
IBMX	Inhibitors: PDE inhibitor (broad spec), adenosineR agonist	37.5
LY-171883	Bioactive lipids: Leukotriene D4 receptor antagonist	2
Gefitinib	Antineoplastic agent	7.47
Hexestrol	Antineoplastic agent; non-steroidal hormone substitute	12.34
Riluzole·HCl	Excitatory amino acid antagonist; anticonvulsant	12.35
Piperacillin	Antibiotic	6.44
Azathioprine	Antimetabolite, antineoplastic, immunosuppressive agent	12.03
5'-N-Ethylcarboxamidoadenosine (NECA)	CNS receptor ligands: adenosine receptor agonist	27.03
Zoledronic acid	Bone density conservation agent	12.25
Sulfadimethoxine	Antiinfective agent	10.75
Ala-Ala-Phe-CMK	Protease inhibitors: Tripeptidyl peptidase II inhibitor	24.52
Camptothecin	Antineoplastic agent; topoisomerase inhibitor	9.58
Gliclazide	Hypoglycemic agent	10.32
Etazolate	Anti-psychotic agent	11.53
Phenamil	Ion channel ligands: Sodium channels	27.26

8-methoxymethyl-IBMX	Inhibitors: phosphodiesterase (PDE1) inhibitor	31.29
AA-861	Lipid biosynthesis: 5-lipoxygenase inhibitor	25.53
Cefepime	Antiinfective agent	6.94
Ampiroxicam	NSAID	7.46
Decoyinine	Inhibitors: lowers GTP levels	29.84
