# Supplementary material for: A Zebrafish Drug-Repurposing Screen Reveals sGC-Dependent and sGC-Independent Pro-Inflammatory Activities of Nitric Oxide
Source: PLoS One. 2015 Oct 7;10(10):e0137286. doi: 10.1371/journal.pone.0137286 (PMC4596872; doi:10.1371/journal.pone.0137286)
Supplement: S2 Table — (PDF) [file pone.0137286.s005.pdf]

S2 Table (Related to Fig 1) Pro-inflammatory compounds

Compound name	Description	Screening concentration [μM]
RIFAMYCIN SV	Antirheumatic agent	4.79
Cyclopiazonic acid	Ion channel ligands: Intracellular calcium	24.77
Nifekalant	Antiarrhythmic agent	8.23
Venlafaxine	Antidepressive agent	12.03
Vinorelbine	Antineoplastic agent	4.28
Ketanserin	Antihypertensive agent	8.43
Gestrinone	Contraceptive agent	10.82
Bromhexine	Antibiotic	8.91
Chlorpheniramine	Histamine H1 antagonist	12.16
Clopidogrel	P2-receptor antagonist	10.38
Tinidazole	Antiinfective agent; antiparasitic agent	13.49
Naloxonazine	Opioid receptor antagonist	5.13
Ticlopidine	Fibrinolytic agent	12.67
Trichloromethiazide	Sodium chloride symporter inhibitor	8.8
Zonisamide	Anticonvulsant, antioxidant	15.72
Azaperone	Neuroleptic sedative	10.19
Dexamethasone	Glucocorticoid	8.5
taxol = paclitaxel	Inhibitors: microtubule stabilizer	9.76
Bupivacaine	Local anesthetic	11.57
Acemetacin	NSAID	8.03
Tobramycin (free base)	Antiinfective agent	7.13
Vindesine sulfate	Antineoplastic agent	3.92
Carbamazepine	Analgesic; anticonvulsant	14.12
Tosufloxacin	Antibiotic	8.25
Trifluperidol	Dopamine antagonist; anti-psychotic	8.15
Fluspirilene	Ion channel ligands: Potassium channels	17.52
Tranylcypromine	MAO inhibitor	25.04
Guaifenesin	Expectorant	16.83
(S)-(-)-Sulpiride	Dopamine D2-receptor antagonist; antipsychotic	9.77
Decamethonium	Muscle relaxant	12.9
Naftopidil·2HCl	Adrenergic alpha1 receptor antagonist	7.18
Nalbuphine	Narcotic	9.33
Yohimbine·HCl	Adrenergic alpha2 receptor antagonist	8.54
Doxofylline	PDE inhibitor	12.53
Haloperidol	Dopamine antagonist; anti-psychotic	8.89
Climbazole	Antifungal agent	11.41
Ondansetron	Anxiolytic agent	11.37
Phenylpropanolamine	Anti-obesity agent	22.06
Esmolol	Adrenergic beta1 receptor antagonist	11.29

H7	Kinase inhibitors: kinase inhibitor	28.6
Neostigmine	AChE inhibitor	14.94
Atovaquone	Antiparasitic agent	9.1
Desloratadine	Histamine H1 antagonist	10.75
Astemizole	Histamine H1 antagonist	7.27
Amprenavir	Anti-HIV agent	6.6
Propidium iodide	Inhibitors: DNA intercalator	12.47
Bopindolol	Adrenergic antagonist	8.77
Ketoconazole	Antiinfective agent, antifungal agent	6.29
Alendronate	Bone density conservation agent	13.44
Finasteride	Alpha5 reductase inhibitor	8.95
Chlormadinone Acetate	Androgen antagonist; contraceptive agent	7.44
Thalidomide	Angiogenesis inhibitor	12.91
Zardaverine	Inhibitors: phosphodiesterase (PDE1/2) inhibitor	31.07
Dextromethorphan	NMDA receptor antagonist	12.29
Estrone	Estrogen	12.34
Toremifene	bone density conservation agent	8.23
Levocabastine	Histamine H1 antagonist	7.31
1-Hexadecyl-2-O-acetyl-glycerol	Bioactive lipids: Blocks DAG activation of PKC	2
Mianserin·HCl	Anti-depressant	11.11
Lomustine	Antineoplastic agent	14.3
Anandamide (18:2,n-6)	Endocannabinoids: Cannabinoid receptor agonist	2
HA1077	Calcium channel blocker	11.45
Tulobuterol	Anti-asthmatic	14.68
5-Aminosalicylic acid	NSAID	21.79
Formestane	Antineoplastic agent	11.03
Praziquantel	Antiinfective agent; antiparasitic agent	10.68
TRIM	Inhibitors: bNOS/iNOS inhibitor	39.27
Furafylline	Enzyme inhibitor	12.82
