# Supplementary material for: A Zebrafish Drug-Repurposing Screen Reveals sGC-Dependent and sGC-Independent Pro-Inflammatory Activities of Nitric Oxide
Source: PLoS One. 2015 Oct 7;10(10):e0137286. doi: 10.1371/journal.pone.0137286 (PMC4596872; doi:10.1371/journal.pone.0137286)
Supplement: S3 Table — (PDF) [file pone.0137286.s006.pdf]

S3 Table (Related to Fig 1) Anti-resolution compounds

Compound name	Description	Screening concentration [μM]
Cycloheximide	Inhibitors: protein synthesis inhibitor	29.62
Nifedipine	Calcium channel blocker; reproductive control agent	9.63
Actinomycin D	Inhibitors: transcription inhibitor	17.94
Cyproheptadine	Histamine H1 antagonist	11.61
Esomeprazole	Anti-ulcer agent	9.66
Cyclopiazonic acid	Ion channel ligands: Intracellular calcium	24.77
Docetaxil	Antineoplastic agent	4.13
Daunorubicin	Antibiotic	6.32
Fluocinolone acetonide	Glucocorticoid	7.37
