# Supplementary material for: A Zebrafish Drug-Repurposing Screen Reveals sGC-Dependent and sGC-Independent Pro-Inflammatory Activities of Nitric Oxide
Source: PLoS One. 2015 Oct 7;10(10):e0137286. doi: 10.1371/journal.pone.0137286 (PMC4596872; doi:10.1371/journal.pone.0137286)
Supplement: S4 Table — (PDF) [file pone.0137286.s007.pdf]

S4 Table (Related to Fig 1) Toxic compounds and compounds with adverse effects

Compound name	Description	Screening concentration [μM]
Miltefosine	Antiinfective agent, antifungal agent	8.18
Quinacrine	Antiinfective agent; antiparasitic agent	8.35
C-PAF	Bioactive lipids: PAF receptor agonist	2
beta-lapachone	Inhibitors: topoisomerase 1 inhibitor	34.39
AG-879	Kinase inhibitors: NGF receptor inhibitor	26.33
Ebselen	Inhibitors: glutathione peroxidase mimetic	30.39
QNZ	Inhibitors: NFkappaB inhibitor	23.38
Calyculin A	Inhibitors: PP1, PP2A inhibitor	0.83
Nigericin	Inhibitors: induces intracellular acidification	11.49
SKF-96365	Ion channel ligands: Calcium channels	22.74
Curcumin	Inhibitors: NFkappaB inhibitor	22.62
Parthenolide	Inhibitors: IkappaB kinase inhibitor	33.56
NPPB	Ion channel ligands: Misc. channels	27.75
4-Amino-1,8-naphthalimide	Inhibitors: PARP inhibitor	39.27
Vinpocetine	Inhibitors: phosphodiesterase (PDE1) inhibitor	23.78
Cantharidin	Inhibitors: PP2A inhibitor	42.47
Cytochalasin B	Inhibitors: F actin capper	17.38
Chelerythrine	Kinase inhibitors: PKC inhibitor	23.92
Flufenamic acid	Ion channel ligands: Potassium channels	29.63
Cytochalasin D	Inhibitors: F actin capper	16.42
Damnacanthal	Kinase inhibitors: p56lck inhibitor	29.53
Nocodazole	Inhibitors: tubulin inhibitor	27.66
Oligomycin A	Inhibitors: Fo ATP synthase inhibitor	10.53
Hinokitiol	Inhibitors: Iron chelator	50.75
Doxorubicin	Inhibitors: topoisomerase II inhibitor, induces apoptosis	14.37
Phorbol 12-myristate 13-acetate	Activators: PKC activator	13.51
FCCP	Inhibitors: mitochondrial uncoupler	32.78
Ro 31-8220	Kinase inhibitors: PKC inhibitor	18.21
RK-682	Inhibitors: VHR phosphatase inhibitor	22.61
Rottlerin	Kinase inhibitors: PKC delta inhibitor	16.13
Nimesulide	Lipid biosynthesis: Cox 2 inhibitor	27.03
Hoechst 33342·3HCl (BisBenzimide)	Inhibitors: DNA minor groove binder	14.83
Ionomycin	Ion channel ligands: Ca++ ionophore	12.27
NSC-95397	Inhibitors: CDC25 phosphatase inhibitor	26.85
staurosporine	Kinase inhibitors: kinase inhibitor - non-selective	17.86

Latrunculin B	Inhibitors: Actin inhibitor	21.07
BAY 11-7082	Inhibitors: Inhibits IKK kinase activation	40.22
Tyrphostin-8	Inhibitors: Calcineurin inhibitor	48.96
tunicamycin	Inhibitors: glycosylation inhibitor	11.6
tyrphostin 9	Kinase inhibitors: PDGF-R tyrosine kinase inhibitor	29.51
LY-83583	Inhibitors: Inhibits NO-activation of guanylate cyclase	33.29
Mastoparan	Activators: activates heterotrimeriuc GTPases	4.94
Gliotoxin	Protease inhibitors: Inhibitor of 20S-proteasome chymotrypsin activity	25.53
U73122	Lipid biosynthesis: PLC inhibitor	17.94
valinomycin	Ion channel ligands: K+ ionophore	7.5
wortmannin	Lipid biosynthesis: PI-3Kinase, other kinases inhibitor	19.45
Shikonin	Inhibitors: Apoptosis inducer, p53 dependent	28.91
CDC	Lipid biosynthesis: 12-Lipoxygenase inhibitor	25.94
