# Supplementary material for: A Zebrafish Drug-Repurposing Screen Reveals sGC-Dependent and sGC-Independent Pro-Inflammatory Activities of Nitric Oxide
Source: PLoS One. 2015 Oct 7;10(10):e0137286. doi: 10.1371/journal.pone.0137286 (PMC4596872; doi:10.1371/journal.pone.0137286)
Supplement: S5 Table — (PDF) [file pone.0137286.s008.pdf]

S5 Table (Related to Fig 1) Injury preventing compounds

Compound name	Description	Screening concentration [μM]
Lomofungin	Antibiotic	10.61
Procaterol·HCl	Adrenergic agonist	10.22
Tolcapone	Antiparkinson drug	12.21
Entacapone	Antiparkinson drug	10.93
Bleomycin	Antibiotic, antineoplastic	2.36
Piroxicam	NSAID	10.07
Ergothioneine	Antioxidant	14.55
Benserazide	Antiparkinson drug	12.97
Methyldopa	Adrenergic alpha2 receptor agonist	15.79
Allopurinol	Xanthine oxidase inhibitor; free radical scavenger	24.51
Fenoldopam	Dopamine agonist	10.93
Levodopa	Dopamine agonist; Antiparkinson drug	16.91
Losartan potassium	Angiotensin II Type 1 Receptor antagonist	7.24
Sulbactam	Antiinfective agent	14.31
Lipoxin A4	Bioactive lipids: Bioactive arachidonic acid metabolite	0.2
Prostaglandin A_1	Bioactive lipids: Bioactive prostaglandin	2
N-Acetyl-S-geranyl-L-cysteine	Bioactive lipids: Negative control for AGGC and AFC	2
Benzamil·HCl	Ion channel ligands: Calcium channels	23.4
Zaprinast	Inhibitors: phosphodiesterase (PDE1) inhibitor	30.72
AG213 (Tyrphostin 47)	Kinase inhibitors: EGF-R tyrosine kinase inhibitor	37.84
AG-490	Kinase inhibitors: JAK2 inhibitor	28.32
B581	Inhibitors: farnesyltransferase inhibitor	17.7
Bestatin	Protease inhibitors: Aminopeptidase inhibitor	27.02
Capsazepine	Ion channel ligands: vanilloid receptor antagonist	22.11
D609	Lipid biosynthesis: PC-PLC inhibitor	39.63
Piroxicam	Lipid biosynthesis: COX1 inhibitor	25.15
GM6001	Protease inhibitors: broad spectrum MMP inhibitor	21.45
Puromycin·2HCl	Inhibitors: protein synthesis inhibitor	15.71
Quercetin·$2H_2O$	Kinase inhibitors: kinase inhibitor (plus other)	24.63
Go6976	Kinase inhibitors: PKC inhibitor	22.02
Histamine	CNS receptor ligands: Histamine receptor agonist	75.01
Indirubin	Kinase inhibitors: GSK-3beta inhibitor	31.77
Piceatannol	Kinase inhibitors: Syk inhibitor	34.13
Juglone	Inhibitors: PIN1 inhibitor	47.84
tamoxifen	Nuclear receptor ligands: estrogen	22.43

	antagonist	
tyrphostin AG-825	Kinase inhibitors: HER-1,2 tyrosine kinase inhibitor	20.96
TPEN	Inhibitors: cell permable heavy metal chelator	19.63
trichostatin-A	Inhibitors: histone deacetylase inhibitor	27.56
