# Supplementary material for: A Zebrafish Drug-Repurposing Screen Reveals sGC-Dependent and sGC-Independent Pro-Inflammatory Activities of Nitric Oxide
Source: PLoS One. 2015 Oct 7;10(10):e0137286. doi: 10.1371/journal.pone.0137286 (PMC4596872; doi:10.1371/journal.pone.0137286)
Supplement: S6 Table — (PDF) [file pone.0137286.s009.pdf]

S6 Table. Transgenic zebrafish lines, oligonucleotide and morpholino sequences

Oligonucleotide name	Target gene	Sequence (5' --> 3')	Experimental use
nos1-fwd	*nos1*	TGGGGTGGAGGATAACAATG	MO specificity RT-PCR
nos1-rev	*nos1*	ACAGCCTTGGTAGGAGAAACTC	MO specificity RT-PCR
nos2a-fwd	*nos2a*	ATGGGAAGACAAGCACAAACC	MO specificity RT-PCR
nos2a-rev	*nos2a*	CATCTGGGGAAGGTGTGATT	MO specificity RT-PCR
nos2b-fwd	*nos2b*	TCCTTGAGGCAAAATTCCAC	MO specificity RT-PCR
nos2b-rev	*nos2b*	GCATTCCTCCATGCTTGTTT	MO specificity RT-PCR
gucy1a3-fwd	*gucy1a3*	GGAGAGGACCATTACGCAAA	MO specificity RT-PCR
gucy1a3-rev	*gucy1a3*	GAAACACCTCCAGACCCAGA	MO specificity RT-PCR
MO*nos1* [1]	*nos1*	TTAATGACATCCCTCACCTCTCCAC	Gene knockdown
MO*nos2a* [2]	*nos2a*	ACAGTTTAAAAGTACCTTAGCCGCT	Gene knockdown
MO*nos2b*	*nos2b*	TGCAATAATACCTCACCTTAACCGC	Gene knockdown
MO*gucy1a3*	*gucy1a3*	ACATCGGACAAACGATTTTACCTCT	Gene knockdown
**Transgenic zebrafish lines**			
Tg(lyz:DsRED2)nz50 [3]			
Tg(-8.0cldnb:lynEGFP)zf106 [4]			

[1] North, T. E., Goessling, W., Peeters, M., Li, P., Ceol, C., Lord, A. M., Weber, G. J., Harris, J., Cutting, C. C., Huang, P., Dzierzak, E., and Zon, L. I. (2009) Hematopoietic stem cell development is dependent on blood flow. Cell 137, 736–748.

[2] Hall, C. J., Flores, M. V., Oehlers, S. H., Sanderson, L. E., Lam, E. Y., Crosier, K. E., and Crosier, P. S. (2012) Infection-Responsive Expansion of the Hematopoietic Stem and Progenitor Cell Compartment in Zebrafish Is Dependent upon Inducible Nitric Oxide. Cell Stem Cell 10, 198–209.

[3] Hall, C., Flores, M., Storm, T., Crosier, K., and Crosier, P. (2007) The zebrafish lysozyme C promoter drives myeloid-specific expression in transgenic fish. BMC Dev Biol 7, 42.

[4] Haas, P., and Gilmour, D. (2006) Chemokine signaling mediates self-organizing tissue migration in the zebrafish lateral line. Dev Cell 10, 673–680.
